# Selective digestive tract decontamination to prevent healthcare associated infections in critically ill children: the PICNIC multicentre randomised pilot clinical trial

**DOI:** 10.1038/s41598-023-46232-7

**Published:** 2023-12-07

**Authors:** Nazima Pathan, Kerry Woolfall, Mariana Popa, Gema Milla de la Fuente, Paloma Ferrando-Vivas, Alanna Brown, Theodore Gouliouris, Lyvonne N. Tume, Robert Shulman, Brian H. Cuthbertson, Isobel Sale, Richard G. Feltbower, John Myburgh, John Pappachan, David Harrison, Paul Mouncey, Kathryn Rowan, the PICNIC study investigators, Charlotte Fulham, Melanie James, Kirsten Beadon, Cat Postlethwaite, Jenny Pond, Antonia Hargadon-Lowe, Jane Cassidy, Ceri Robbins, Phil Milner, Buvana Dwarakanathan, Joana Gomes De Queiroz, Esther Daubney, Deborah White, Peter Davis, Laura Dodge, Francesca Moody

**Affiliations:** 1 University of Cambridge, Cambridge, UK; 2 Cambridge University Hospitals NHS Foundation Trust, Cambridge, UK; 3 University of Liverpool, Liverpool, UK; 4 Intensive Care National Audit and Research Centre, London, UK; 5 University College London, London, UK; 6 Edge Hill University, Lancashire, UK; 7 Sunnybrook Health Sciences Centre, Toronto, Canada; 8 Lay representative, Unaffiliated, UK, UK; 9 University of Leeds, Leeds, UK; 10 The George Institute for Global Health, Sydney, Australia; 11 University of Southampton, Southampton, UK; 12 John Radcliffe Hospital, Ocxofrd, UK; 13 Birmingham Children’s Hospital, Birmingham, UK; 14 St George’s University Hospital, London, UK; 15 University Hospitals Bristol, Bristol, UK

**Keywords:** Policy and public health in microbiology, Clinical microbiology, Antibiotics

## Abstract

Healthcare-associated infections (HCAIs) are a major cause of morbidity and mortality in critically ill children. Data from adult studies suggest Selective Decontamination of the Digestive tract (SDD) may reduce the incidence of HCAIs and improve survival. There are no data from randomised clinical trials in the paediatric setting. An open label, parallel group pilot cRCT and mixed-methods perspectives study was conducted in six paediatric intensive care units (PICUs) in England. Participants were children (> 37 weeks corrected gestational age, up to 16 years) requiring mechanical ventilation expected to last for at least 48 h. Sites undertook standard care for a period of 9 weeks and were randomised into 3 sites which continued standard care and 3 where SDD was incorporated into infection control practice for eligible children. Interviews and focus groups were conducted for parents and staff working in PICU. 434 children fulfilled eligibility criteria, of whom 368 (85%) were enrolled. This included 207 in the baseline phase (Period One) and 161 in the intervention period (Period Two). In sites delivering SDD, the majority (98%) of children received at least one dose of SDD and of these, 68% commenced within the first 6 h. Whilst admission swabs were collected in 91% of enrolled children, consent for the collection of additional swabs was low (44%). Recruited children were representative of the wider PICU population. Overall, 3.6 children/site/week were recruited compared with the potential recruitment rate for a definitive cRCT of 3 children/site/week, based on data from all UK PICUs. Parents (n = 65) and staff (n = 44) were supportive of the aims of the study, suggesting adaptations for a larger definitive trial including formulation and administration of SDD paste, approaches to consent and ecology monitoring. Stakeholders identified preferred clinical outcomes, focusing on complications of critical illness and quality-of-life. A definitive cRCT in SDD to prevent HCAIs in critically ill children is feasible but should include adaptations to ecology monitoring along with the dosing schedule and packaging into a paediatric specific format. A definitive study is supported by the findings with adaptations to ecology monitoring and SDD administration.

*Trial Registration:* ISRCTN40310490 Registered 30/10/2020.

## Introduction

Critical illness affects immune competence and in those requiring prolonged organ support, this, along with the presence of invasive devices such as urinary catheters, vascular lines and endotracheal tubes places them at risk of secondary infection. In children with severe or prolonged critical illness, healthcare-associated infections (HCAIs) are a major cause of morbidity and mortality, with a prevalence of 7–14%^1–5^. A key source of HCAI’s are the opportunistic microorganisms residing in the oral cavity and gastrointestinal tract^4,5^. Evidence from adult intensive care studies suggests that using Selective Decontamination of the Digestive tract (SDD) alongside standard infection control measures may reduce mortality and ventilator-associated pneumonia (VAP), although results are mixed in terms of clinical benefit^6–8^.

The effect of SDD on antimicrobial resistance in treated patients and the unit ecology is not well described but remains an important concern for clinicians in the critical care setting^8–11^. In an adult trial of SDD, ecology assessment in over 8500 patients found significant reduction in positive blood cultures and cultures of antibiotic-resistant organisms with no significant increase in new *Clostridiodes difficile* infections in SDD treated patients compared to standard care^12^. A meta-analysis of 32 randomised adult clinical trials including 24,389 participants suggested use of SDD compared with standard care or placebo was associated with reduced hospital mortality^13^. The estimated risk ratio (RR) for mortality for SDD compared with standard care was 0.91 (95% credible interval [CrI], 0.82–0.99; I2 = 33.9%; moderate certainty) with a 99.3% posterior probability that SDD reduced hospital mortality.

Other meta-analyses suggest SDD is not associated with the development of antimicrobial resistance in patients in the ICU, although the effect of decontamination on ICU-level antimicrobial resistance rates is understudied^13–16^

Evidence for the use of SDD in the Paediatric Intensive Care (PICU) population is limited to single centre and small observational studies^17^. In this pilot cluster randomised trial (cRCT), we explored the feasibility of conducting a definitive, multicentre cRCT comparing SDD to standard infection control in the PICU setting with the following objectives: (1) willingness and ability to recruit eligible patients; (2) adherence to the SDD intervention; (3) acceptability of the definitive cRCT; (4) estimation of recruitment rate; (5) understanding of potential clinical and ecological outcome measures^18^.

The underlying hypothesis is that SDD is superior to standard infection control measures in preventing HCAIs in children expected to require at least 48 h of mechanical ventilation.

## Methods

### Trial design and oversight

The PICnIC trial was an open-label multicentre RCT in infants and children admitted to one of six participating PICUs (Birmingham, Bristol, Cambridge, John Radcliffe Hospital Oxford, Southampton and St George’s University Hospital London) and an integrated mixed methods study. Sites were selected for geographical spread across England with common configurations for UK PICUs, including a mix of general and cardiac critical care cases within academic, general and standalone children’s hospital settings.

The trial was co-ordinated by the Intensive Care National Audit & Research Centre (ICNARC) Clinical Trials Unit (CTU), and the overall study sponsored by the University of Cambridge and Cambridge University Hospital Biomedical Research Centre.

The study was approved by the Health Research Authority (IRAS number: 239324) and registered on the ClinicalTrials.gov database (ISRCTN40310490) and all methods were performed in accordance with the approved study protocol. The full study protocol has been previously published, see also Supplementary data^18^.The pilot, parallel group cRCT was run over a period of 18 weeks from 18th September 2021. In Periods One (standard care) and Two (standard care or SDD enhanced infection control), all children expected to need invasive mechanical ventilation for at least 48 h were eligible for inclusion in the study and had nasopharyngeal and rectal swabs on admission (research without prior consent) and then twice weekly (with consent). Three week-long periods of unit ecology surveillance were undertaken in week 1 (pre-trial), week 10 (transition period) and week 20 (post trial) with swabs taken from all children admitted to the PICU during these weeks regardless of ventilation status.

### Trial population and eligibility criteria.

Children who were at least 37 weeks corrected gestational age up to 16 years of age were eligible for inclusion if they were receiving, and were expected to remain on, mechanical ventilation for at least 48 h from the time of screening. Exclusion criteria included known allergy, sensitivity or interaction to polymyxin E (colistin), tobramycin or nystatin, pregnancy or where death was perceived as imminent (futility).

### Screening, randomisation and consent

The CTU team regularly reviewed screening logs, which contained details about patient ineligibility criteria (i.e., met one or more exclusion criteria) or reasons for eligible patients not being recruited such as limited research capacity, missed patients, site being near or reached recruitment target and gastro-intestinal contra-indications to enteric medication).

Randomisation of sites for SDD intervention was undertaken on week 5 by the trial statistician using computer-based randomisation. Sites were later informed of the outcome on week 8.

### Consent

As with previous studies of SDD in adults^12,13^, informed consent for routine microbiology and administration of SDD in sites randomised to intervention was not required as these were incorporated into routine PICU infection control procedures (unit wide implementation is key for the validity of SDD as an ecology-modifying treatment in the ICU setting). Informed parental consent was however sought for additional study-specific swabs to monitor ecology over the course of illness and for parents of enrolled children to take part in an interview.

### Trial interventions

The intervention selected was the addition of Selective Digestive tract Decontamination (SDD) to the standard infection control strategy of the participating PICU. The intervention consisted of three topical antimicrobial agents, colistin, tobramycin and nystatin, prepared according to international standards for Good Manufacturing Procedures and manufactured in a Therapeutic Goods Administration-approved facility, Verita Pharma®, Sydney, under licence from the George Institute for Global Health, Sydney. The SDD preparations were manufactured specifically for the SuDDICU randomised controlled trial conducted in Australia and Canada between 2017 and 2023^12^. The SDD preparations were imported under temperature-controlled conditions to the UK for this pilot trial. SDD paste (0.5 g) and suspension (2.5-10 ml) containing polymyxin E (colistin), tobramycin and nystatin were administered six-hourly to all eligible patients for the duration of invasive mechanical ventilation according to the study protocol^18^. The intervention was restarted if patients were subsequently re-intubated (either during this PICU admission or readmission to PICU from another inpatient area) during the treatment period.

### Ecology monitoring

Nasopharyngeal and perineal swab samples were taken from all patients at admission as part of routine admission infection control screening procedures. Where consent was obtained, further swabs were taken twice weekly or after 48 h if the child remained on PICU during the ecology week.

### Data collection

Baseline data was collected at critical care admission, including age (years), sex (male, female), weight (kg), ethnic group, disease severity (admission Paediatric Index of Mortality 3 (PIM3) score^19^) and primary clinical diagnosis. To inform the selection of patient-centred primary and secondary outcome measures in a definitive cRCT, a range of clinical outcome measures were collected, including healthcare associated infection, positive microbiology swabs or samples, durations of invasive ventilation, PICU and hospital stay, survival status (at hospital discharge and at 30 days post enrolment).

### Outcome measures and sample size estimation

As a feasibility study to test whether the protocol could identify and recruit eligible patients, no primary outcome was selected to compare standard care and intervention groups. Instead, sample size was determined by the number needed to test critical parameters to a necessary degree of precision. Based on available data from the national Paediatric Intensive Care Audit Network (PICANet), it was anticipated that the participating sites would screen approximately 4.5 eligible children per week, therefore the anticipated recruitment rate was 3 children per PICU per week providing a total of approximately 324 children in 18 weeks, of which 90 would receive the intervention.

### Statistical analysis

The analyses were conducted using Stata/MP version 16.1 (StataCorp, Texas). All patients were included with the intention to treat (ITT) population, excluding only those who withheld or withdrew consent to data collection. Children were analysed according to the group they were randomised to (based on site and date of recruitment), irrespective of whether the treatment allocated was received.

Potential outcome measures were summarised using counts and percentages (for binary outcomes), median and IQR (for all continuous outcome) and means and standard deviations (length of stay and duration of treatment) and reported by intervention group and study period.

To account for cluster randomisation, multilevel logistic or generalised linear regressions were used to estimate potential treatment effects. The effect estimate was calculated as the interaction between treatment group and study period, and reported as either odds ratio with 95% CI (binary outcomes) or difference in means with 95% CIs (continuous outcomes).

As this was a pilot cRCT and not powered to detect differences in outcomes, analyses are treated as exploratory and were mainly descriptive. P values are not calculated or quoted.

### Parent and staff perspectives

To explore perspectives of key stakeholders on the acceptability and design of a definitive SDD cRCT, we invited parents of children in participating units to complete a questionnaire and/or participate in an interview approximately a month after leaving hospital. We also invited staff involved in the pilot trial to take part in a focus group at the end of recruitment. Interviews and focus groups were audio recorded after consent was obtained. The methodology for qualitative data analysis is outlined in supplementary Table 1.

Qualitative thematic analysis was interpretative and iterative and informed by the constant comparative approach. NVivo 10 software (QSR International, Warrington, UK) was used to assist the coding of data. Survey data were analysed using SPSS for descriptive statistics. Qualitative and quantitative data were analysed separately and then synthesised through constant comparative analysis.

### Ethical approval

The study was approved by the UK Health Research Authority (IRAS number: 239324).

## Results

### Patient characteristics for the pilot cRCT

**Figure 1:**
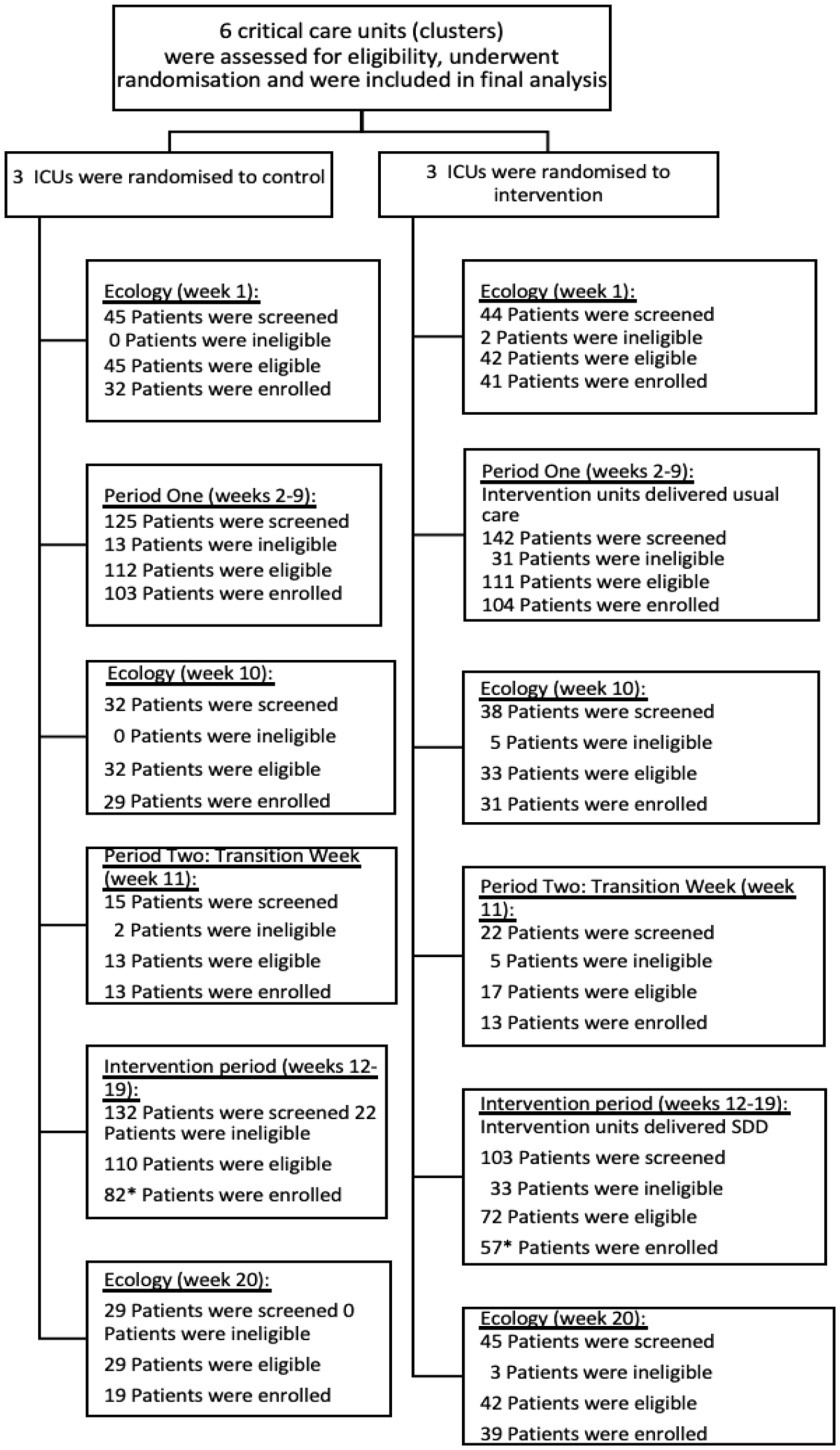
Consort diagram.

Enrolment across the baseline, intervention and ecology study periods are shown in Fig. 1.

Patients were well balanced across treatment groups and time periods (Table 1). The mean age of patients was similar in Periods One (33.3 months in the usual care sites and 31.5 months in the intervention sites), and Two (28.0 months in the usual care sites and 25.0 months in the intervention sites). Over 63% of the patients were male. The PIM3 score was similar across treatment groups, with a median predicted mortality risk of 2%. The most common primary diagnostic group of the recruited children was respiratory (43%), followed by cardiac (23%).

**Table 1:** Patient characteristics.

Variables	Recruited patients			
	Intervention sites		Control sites	
	Period one	Period two	Period one	Period two
	N = 104	N = 56	N = 103	N = 82
Age at admission (months)				
Median (IQR)	7 (48)	5 (18)	7 (43)	5 (25)
Mean (SD)	31.5 (46.5)	25.0 (45.3)	33.3 (52.5)	28.0 (49.0)
Age category at admission				
< 1 year	55/101 (54.5%)	34/55 (61.8%)	55/100 (55.0%)	53/79 (67.1%)
1 years	13/101 (12.9%)	9/55 (16.4%)	15/100 (15.0%)	5/79 (6.3%)
2–4 years	8/101 (7.9%)	3/55 (5.5%)	9/100 (9.0%)	7/79 (8.9%)
5–9 years	15/101 (14.9%)	5/55 (9.1%)	9/100 (9.0%)	6/79 (7.6%)
10–16 years	10/101 (9.9%)	4/55 (7.3%)	12/100 (12.0%)	8/79 (10.1%)
Sex				
Male	58/101 (57.4%)	38/55 (69.1%)	64/100 (64.0%)	49/79 (62.0%)
Female	43/101 (42.6%)	17/55 (30.9%)	36/100 (36.0%)	30/79 (38.0%)
Ethnic category				
Asian	12/101 (11.9%)	8/55 (14.5%)	5/100 (5.0%)	4/79 (5.1%)
Black	9/101 (8.9%)	1/55 (1.8%)	2/100 (2.0%)	3/79 (3.8%)
Chinese	0/101 (0.0%)	1/55 (1.8%)	0/100 (0.0%)	0/79 (0.0%)
Mixed	6/101 (5.9%)	1/55 (1.8%)	3/100 (3.0%)	6/79 (7.6%)
White	55/101 (54.5%)	36/55 (65.5%)	63/100 (63.0%)	41/79 (51.9%)
Other	8/101 (7.9%)	3/55 (5.5%)	2/100 (2.0%)	0/79 (0.0%)
Unknown	11/101 (10.9%)	5/55 (9.1%)	25/100 (25.0%)	25/79 (31.6%)
PIM3 predicted risk of PICU mortality (%)				
Median (IQR)	4 (7)	3 (5)	2 (4)	2 (5)
Mean (SD)	5.8 (7.5)	6.3 (12.6)	4.2 (7.8)	5.7 (11.9)

### Parent and staff characteristics for the qualitative study

A total of 65 parents of randomised children completed the survey. This included parents who consented to ongoing participation in the trial (n = 63) and some who declined consent (n = 2). 15 were parents (23%) of children in the intervention group (SDD), 24 (36%) in the control (standard care) group, and 24 (36%) during ecology week. The survey was completed by 44 staff members, with 23 (52%) being nurses and 19 (43%) doctors, representing a total of 11 UK PICUs. Six focus groups were conducted, which involved 26 staff members, with 17 (65%) being nurses, 8 (30%) being doctors, and 1 (5%) being a pharmacist.

### Objective 1: Willingness and ability to recruit eligible patients

**Figure 2:**
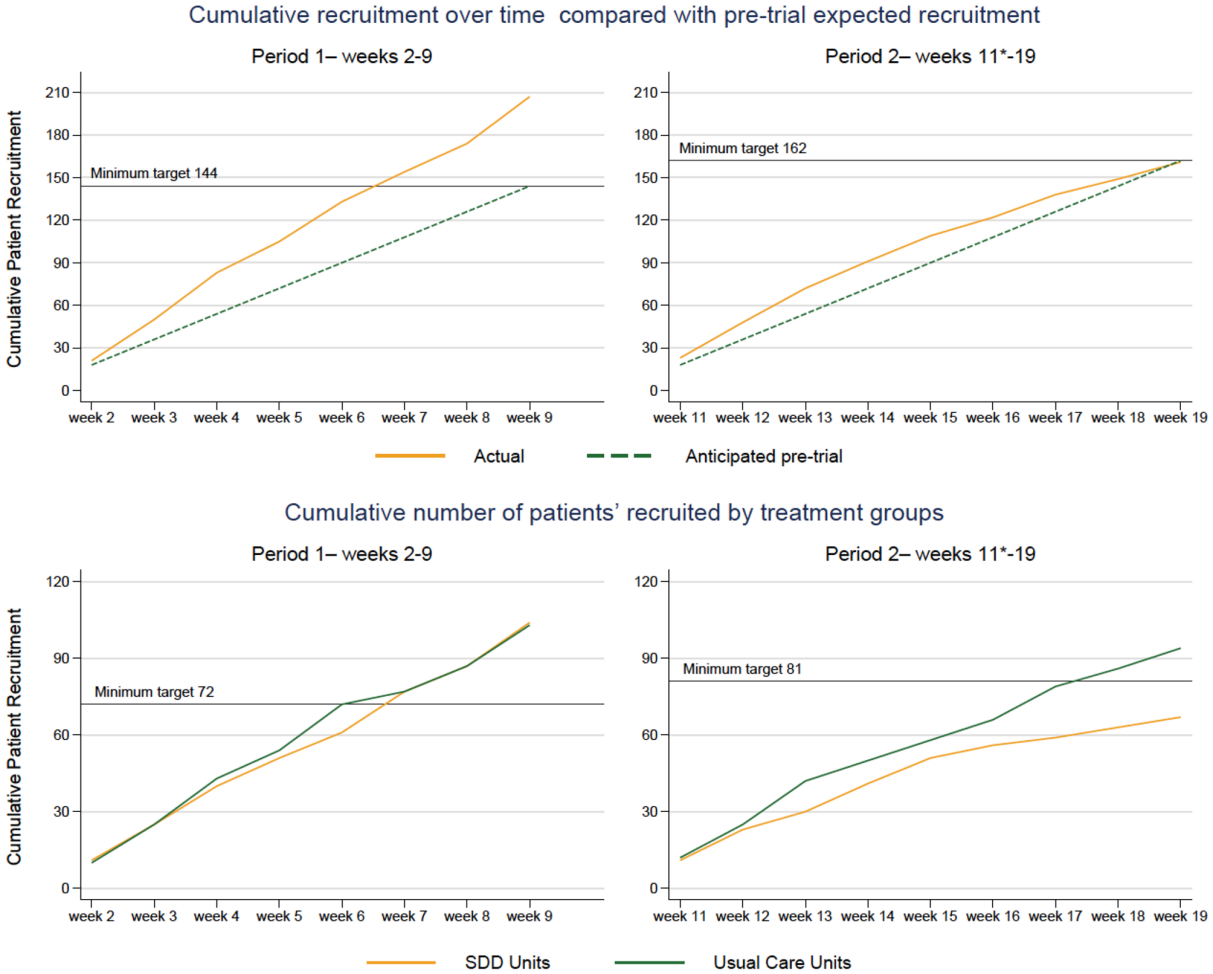
shows cumulative recruitment in the two periods of the study (period 1, left and 2, right). The top panels show actual (orange) versus anticipated (green) enrolment. The bottom panels show enrolment in each period based on the unit randomisation in period 2 (standard care, green; SDD-enhanced infection control, orange).

Between 19 September 2021 and 13 February 2022, 539 children were screened for inclusion into the baseline and intervention phases of the trial (Periods one and two), of which 433 (80.3%) were eligible and 368 (84.9%) of these were enrolled (Fig. 2).

This was higher than the pre-trial expected recruitment of 306 children across the course of Periods One and Two. In the three ecology weeks a total of 233 children were screened, 223 (95.7%) eligible and 191 (85.6%) were enrolled.

Issues with eligible children being missed were due to difficulties in staff knowing whether children would be ventilated for 48 h, as per inclusion criteria: *“We haven’t got a crystal ball, we don’t know.”* (P10, FG3, intervention group, research nurse). Some sites screened more than once up until the 48-h window to overcome the risk of not enrolling children who were not originally expected to be ventilated for > 48 h but then required it. Staffing issues due to the COVID-19 pandemic and not remembering to rescreen after the point of admission impacted upon recruitment. Concurrent recruitment of multiple studies and processing of samples in labs that were already overburdened seemed to intensify the issues.

### Objective 2: adherence to the SDD intervention

#### Adherence to SDD intervention

After excluding transition week, a total of 56 children were recruited in the intervention sites, and 55 of them (98%) received SDD treatment (Table 2). Around 68% of eligible children received SDD within the first 6 h as per protocol. Of the expected doses, 9.2% of the oral paste and 9.1% of the gastric Suspension were not administered. The main reasons were being ‘nil by mouth’ (29.5% and 31.1%) and ‘dose missed’ (26.2% and 24.6%).

**Table 2:** Adherence to SDD intervention.

	SDD treatment method		
	Any	Oral paste	Gastric Suspension
Patients			
Enrolled, n	56		
Received SDD, n (%)	55 (98.2)		
Received SDD within 6 h of enrolment, n (%)	38 (67.9)		
Doses			
Total expected^1^, n		1330	1330
Total administered, n (%)		1208 (90.8)	1209 (90.9)
Total not administered, n (%)		122 (9.2)	121 (9.1)
Median (IQR) per patient		14.0 (9.0, 32.0)	14.0 (9.0, 32.0)
Reasons for doses not given			
Not applicable, n (%)		13 (10.7)	4 (3.3)
Nil by mouth, n (%)		36 (29.5)	38 (31.1)
Unable to administer via prescribed route, n (%)		4 (3.3)	5 (4.1)
Dose missed, n (%)		32 (26.2)	30 (24.6)
Omitted on clinician's instruction, n (%)		6 (4.9)	6 (4.9)
SDD not available, n (%)		10 (8.2)	13 (10.7)
Prescription issues, n (%)		9 (7.4)	14 (11.5)
Parent decision, n (%)		11 (9.0)	10 (8.2)

n: Number of patients or doses; %: Percentage of patients or doses.; ^1^ Patient ventilated (based on CRF data).

Overall, focus group participants were mainly positive about the SDD intervention and its use in the proposed trial.

*“I’m very aware of healthcare-associated infections, and I think anything that can reduce that has to be good. I would love this pilot to work and this trial to go ahead, to see whether there’s the evidence to back up my hope for it to be of use in reducing healthcare-associated infections. I think it has to be in a way which is acceptable clinically and for families.”* (P05, FG2 part 2, intervention site, PI/doctor)

*I was interested in the fact that it can reduce the amount of antimicrobials used in that patient, but also in other patients on the unit. That's what interested me quite a bit was the effect on the microbiological sort of flora* (sic) *within the unit.”* (P18, FG5, control group, PIC consultant).

Challenges identified by staff who administered the SDD intervention included: storage of drug, logistics and packaging, and look of the paste.

*“The fact that each of these children had to have their own kit, which seems wasteful. …Obviously, the nurse that’s looking after the patient has got other things to do, she’s now to got to make this drug up in order to give it to the patient. If it was just a bottle in the cupboard she would have done it, but because of the way it’s been dispensed in the fact that each patient has to have their own kit.” (P10, FG3, intervention site, research nurse)*

Parents were supportive of a future trial that include the SDD intervention, however, they expressed that they would want the research team to explain better what medication their child is being given.

*“Yes, I definitely think that’s important information, it should be clarified. Especially because it’s not typical standard of care, do you know what I mean? Like, I think you should have all the knowledge, as a parent, going into it, exactly. Especially what is being put into your child; you should know.” (P21, mother, SDD)*

#### Adherence to ecology monitoring

Most patients (91–99% across periods) had swab samples taken at admission to the PICU. However, study-specific ecology was impacted by a low rate of consent (44% of enrolled children) for ongoing (twice-weekly) screening swabs. When consent was obtained, adherence to the sampling regimen was good with samples collected for more than 90% of eligible patients at each timepoint. There was good collection of all routine microbiology cultures and antimicrobial administration during the PICU stay.

### Objective 3: acceptability of the definitive cRCT

Using a five-point Likert scale ranging from very unacceptable to very acceptable, verbal responses and handset voting suggested that staff involved in the PICnIC pilot trial sites found the proposed trial acceptable. All (n = 26) stated they would be interested in participating in the proposed PICnIC trial and no staff (ranging from senior used the voting poll system (n = 24) felt the trial was unacceptable, most indicating it was acceptable (17/24, 71%), or very acceptable (4/24, 17%), while a couple of respondents (2/24, 8%) were neutral.

“I think acceptable. I would say acceptable with, clearly, the feedback* we have given. It needs a bit of finetuning.”* (P01, FG1, intervention site, research nurse)

All parents who completed the questionnaire (65/65, 100%) and were interviewed (23/23, 100%) stated that the proposed PICnIC cRCT was acceptable to conduct. This included parents who declined consent for some aspect of the trial. Having the option to decline certain aspects of trial involvement, such as additional samples, appeared to make the pilot trial more acceptable to parents.

Amongst parents, all of them indicated they considered a definitive trial of SDD in the UK PICU setting acceptable.

*“I said to the nurse at the time as well when I read it, ‘You should definitely roll this out into different intensive care units around the UK or further afield’. Like I say, it can only benefit.”* (P9, father, SDD).

*“It just makes sense to do the study across the whole of the UK, increase the accuracy of the study. Yes, I think it’s a good idea.”* (P15, Father, Control).”

Parents viewed the intervention as non-invasive and were generally satisfied with the provision of information about the nature of the trial, perceiving a low risk from their child’s inclusion in the study.

Some parents (2 control, 4 intervention) hoped or held the misconception that their child would directly benefit from the study:

*“From a point of having it, being in his system, there, ready, it’s just another thing, isn't it? It’s like another protective layer almost, for him to have*.” (P23, mother, SDD).

#### Acceptability and understanding of randomisation

Overall, parents acknowledged the importance of randomisation in a study such as PICnIC and trusted that the practitioners would “*treat the study in an ethical manner*” (P15, father, control).

Two parents thought the question of randomisation was hard to answer, as it would depend on how sick the child is and whether the benefits outweigh the risks.

*“For me, personally, if my son was really very ill, which he was at one point, to find out that there could be a medication out there that could help him to not get worse by catching an infection, then I would want him to have it.”* (P5, mother, Period One control).

Parents in both control and intervention groups wanted to know more about the actual intervention, rather than the trial arm the child had been randomised to.

“I don't think it’s as important as explaining what the actual treatment involves and what that means for your child and what the drawbacks could be to the treatment actually. I think that’s more important than knowing whether your child is actually getting the treatment or not for it.” (P7, father, SDD)

*“Maybe it’s probably worth saying to them, “Look, this is why we’re giving this to your child because everyone on this unit we’d like to give it because we’re comparing it to another unit who is not having it to see the results.” Yes, maybe we could have been told that information*.” (P16, father, control)

#### Parental understanding and acceptance of SDD intervention

While parents had a rough understanding of the study aims, most of them were not aware of what the SDD medication was.

Only one parent was concerned about the child being given an antimicrobial.

*“Oh my God, basically stripping the gut of stuff.”* (P11, mother, Period One control). Their aversion to their child receiving the SDD medication was based on previous experience of PICU, when the child had experienced severe adverse events as a result of being given antimicrobials. However, they were open to persuasion if given the right information

“*It’s difficult I suppose, it depends on how you pitch it anyway doesn’t it? […] if I think my son is at greater risk because he’s more vulnerable…then I’m open to persuasion.”* (P11, mother, Period One control)

#### Acceptability of sample collection and the importance of being informed

The majority of parents thought that it was acceptable to collect routine samples as “*they're taking them anyway”* (P3, mother, control) yet many stated that consent should be sought prospectively for sample collection.

*“I think parents would want to know and would want to give consent on providing samples. Obviously, I guess, it’s all about the protection of their child. Knowing where the blood samples are going, who are they going to, what the bloods are going to be used for.”* (P2, mother, ecology)

Importantly, all parents who completed the questionnaire (65/65, 100%) and were interviewed (23/23, 100%) were satisfied with the approach to recruitment and consent (Tables 3 and 4) stating that the proposed PICnIC cRCT was acceptable to conduct.

**Table 3:** sample size calculations for a definitive cRCT.

	Study outcome (% or mean)	Relative risk/effect sizes SD	Absolute difference	Clusters per arm (sample size per arm)
Healthcare associated infection	11.8%	0.8	2.4%	434 (82,460)
	11.8%	0.6	4.4%	120 (22,800)
Any positive microbiology result	59.7%	0.8	11.9%	53 (10,070)
	59.7%	0.6	23.9%	13 (2,470)
Duration of invasive ventilation (days)	7.6	0.2 SD	1.6	12 (2,280)
	7.6	0.3 SD	2.4	5 (950)
Days alive and free of mechanical ventilation at 28 days	19.5	0.2 SD	1.6	12 (2,280)
	19.5	0.3 SD	2.4	5 (950)
Length of PICU stay (days)	10.1	0.2 SD	2.0	12 (2,280)
	10.1	0.3 SD	3.2	5 (950)
Length of hospital stay (days)	23.3	0.2 SD	5.4	8 (1,520)
	23.3	0.3 SD	8.1	4 (760)
PICU mortality	7.3%	0.8	1.5%	290 (55,100)
	7.3%	0.6	2.9%	70 (13,300)
Hospital mortality	9.4%	0.8	1.9%	179 (34,010)
	9.4%	0.6	3.8%	40 (7,600)
30-day mortality	7.7%	0.8	1.54	182 (34,580)
	7.7%	0.6	3.1%	38 (7,220)

**Table 4:** Characteristics of potential outcome measures among all patients receiving standard care.

Outcome measure	Patient receiving standard care*	Intra class correlation (ICC)
Healthcare associated infection, n (%)	34 (11.8) [288]	0.316 (0.060, 0.771)
Any positive microbiology result	172 (59.7) [288]	0.374 (0.129, 0.707)
Duration of invasive ventilation (days)	7.6 (7.9) [289]	0.012 (0.001, 0.177)
Days alive and free of ventilation to day 28	19.5 (8.1) [280]	0.018 (0.001, 0.188)
Length of PICU stay (days)	10.1 (10.6) [288]	0.014 (0.001, 0.164)
Length of hospital stay (days)	23.3 (26.9) [284]	0.006 (0.000, 0.296)
PICU mortality	21 (7.3) [288]	0.099 (0.007, 0.629)
Hospital mortality	27 (9.4) [286]	0.068 (0.002, 0.684)
30-day mortality	22 (7.7) [286]	0.041 (0.000, 0.803)

*Patients recruited at Control sites throughout Period One and Two, and at Intervention sites during Period One.

### Objective 4: estimation of recruitment rate

#### Characteristics of participating sites

Overall, the sites participating in the study were a representative mix of small and large PICUs with a broad case mix of cardiac and general admissions drawn from across the NHS England geographical region with characteristics that were similar in the 6 sites to the wider PICU admission profile in the UK (Supplementary Table 2).

#### Characteristics of participating patients

Children who were recruited to the PICnIC study were representative of similar potentially eligible patients (ventilated 3 days or more) in the study PICUs and all UK PICUs but were more likely to be male and with a primary diagnosis of infection when compared with all UK PICUs (Supplementary Table 3).

#### Statistical approach to a definitive clinical trial of SDD in the PICU setting

The potential recruitment rate for a future definitive cRCT was 3 children/site/week and was estimated by combining a potentially eligible population of 1730 children (estimated from nesting the screening log data from participating PICUs with the national UK PICU data from PICANet) and the overall proportion of eligible children recruited of 85%. This is very similar to the pre-trial estimated rate value of 3.0. The number of eligible children identified from screening logs in the six recruiting PICUs (434) was very similar to the estimate of potentially eligible children from PICANet for these PICUs (464).

Assuming a parallel arm cluster-randomised design with a baseline period, the number of clusters per arm and overall sample size required to detect alternative treatment effects with 90% power and a significance level of 0.05 are shown in Table 4. The number of children per cluster is set at 190 (based on one year of recruitment, including the baseline period), using the ICC observed and mean/proportion among all patients receiving usual care during the pilot cRCT.

The only binary outcome with sufficient frequency to be potentially feasible was any positive microbiology result; however, a detectable difference in this outcome would need to be greater than would be delivered by prevention of all HCAIs. Suitable patient-centred outcomes include scale variables such as duration of mechanical ventilation or days alive and free of mechanical ventilation or length of PICU stay.

Although the study was not powered to compare outcomes between groups, a summary of the potential outcome effects is shown in Supplementary Table 3 to allow consideration of plausible ranges of treatment effects for a definitive trial. As anticipated for a small pilot study, there were not significant differences between the groups in any of the potential outcomes. A summary of the outcomes by treatment group and time period are shown in Supplementary Table 4.

### Objective 5: understanding of potential clinical and ecological outcome measures

Overall, patient-centred potential outcomes measures had an excellent completion rate, which was similar between groups with a range between 96.3 and 100%.

Characteristics of the patient-centre potential outcomes among all patients receiving standard care (all children in control PICUs and children in intervention PICUs in Period One) are reported in Table 4.

#### Parental perspectives on clinical outcomes in a definitive cRCT

**Figure 3:**
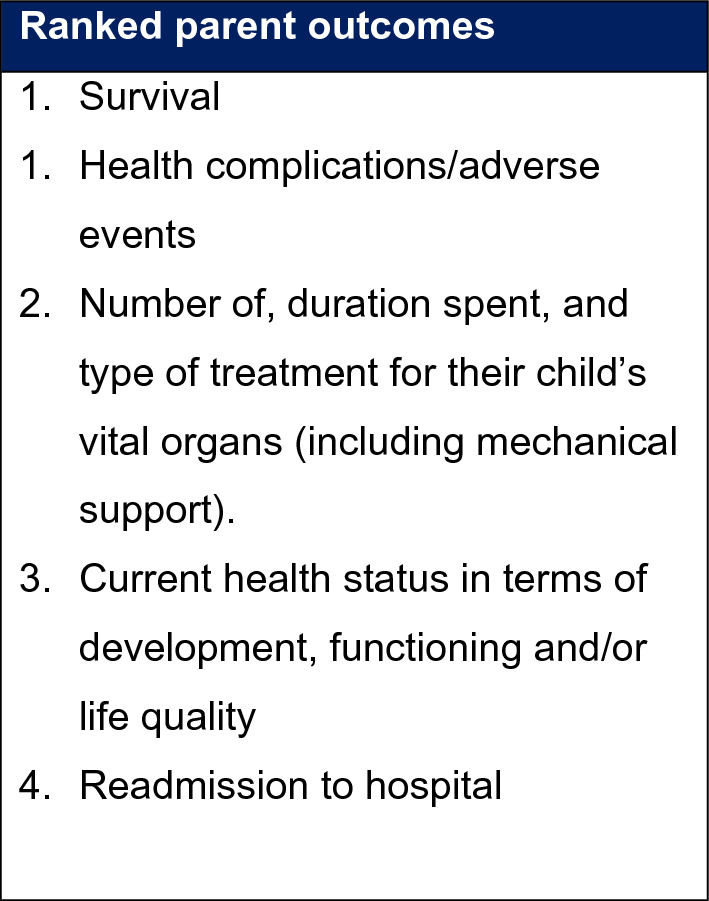
Parent Centred Outcomes for the proposed trial.

Parents were asked to review the list of outcomes that had been sent to them prior to the interview. In the few cases in which parents did not have access to the materials, a definition of each outcome was read to them, including an explanation about why it is important to explore parents’ perspectives about important outcomes (Fig. 3).

Most parents hoped that their child’s quality of life will improve i.e. “*getting back eating; obviously, not having relapses; I think getting back to where she was, developmentally, before she got poorly.”* (P19, father, control), that “*it would reduce the risk of an infection and a need for further intervention”* (P02, mother, ecology).

Most parents prioritised outcomes that were included in the list they were provided with, such as reduced likelihood of complications or infections, “*less complications because they received the SDD, then I think those would be priority.”* (P12, mother, control).

“So, from a parent’s perspective, I’m hoping that this study drives an outcome where secondary infections are less common in children receiving intensive care treatment.” (P07, father, SDD)

Parents were then asked to rank the outcomes listed order of importance from the list provided. The top prioritised outcomes can be seen in Box 1. Survival and health complication/adverse events were ranked joint as most important for the majority of parents.

Although many did not initially mention it, the majority of parents, when prompted, considered survival an important outcome. “*I don’t know, the outcome of survival…I try not to think about that really.”* (P9, father, SDD).

Other outcomes associated with health complications included infections acquired in the ICU, tolerance of the intervention, the number of specific treatments their child received, and looking and behaving like normal self, i.e., an overall feeling of return to health or normality.

## Discussion

The pilot cRCT was representative, in terms of sites and patients when compared with the whole UK PICU population and our analysis suggests a definitive trial is feasible. Several design modifications would need to be included in a definitive cRCT to ensure that the efficiency of trial processes is maximised.

The cluster randomisation model was effective and supported by staff and parents. PICUs were able to be randomised into groups which were relatively evenly split in terms of unit size, and patient characteristics including age, ethnicity and admitting diagnosis.

The contracted nature of the pilot cRCT meant that staff had to adapt practice more quickly than would be expected in a definitive cRCT, where a more prolonged and intensive support could be planned and instituted.

The potential recruitment rate for a future definitive cRCT trial is similar to the pre-trial estimated rate value of 3 per site per week. One site from the intervention group closed to recruitment early due to reaching their recruitment target, which could have an impact in a definitive cRCT. Even though there are limited PICUs in the UK, the sample size simulations indicated that there are sufficient available clusters to adequately power a definitive cRCT on patient centred clinical outcomes, including health care acquired infection and days alive and free from ventilation.

Each of the study periods (e.g. ecology, transition, intervention) need to be long enough to embed the new processes into clinical practice and the timeline. A definitive trial would need to ensure contracting of sites required enrolment of patients for the full period of the cRCT.

Screening and recruitment processes worked efficiently, with the majority of eligible patients enrolled into the study across the ecology periods and Periods One and Two. Some eligible patients may have been missed due to the subjective estimate of whether the patient was likely to be extubated within the next 48 h.

When comparing against the potentially eligible patients within the participating sites, patients enrolled were representative in terms of age and ethnicity. However, the need for translated material potentially limited inclusion of those parents who did not speak English.

Sites implemented the consent model inconsistently, especially early in the pilot cRCT, with a number of parents not being approached for additional samples or data collection. Parents did not object to the consent process, as long as additional samples were only taken with consent. This was evident, as consent for routine data collection was good, but there was a number of refusals for collection of additional samples.

Adherence to the SDD intervention was high, with SDD commencing in a timely manner in most children. The time-critical nature of the study intervention made delivery challenging and has resource implications. There appeared to be subtle differences in how staff described the nature of the intervention at sites administering SDD, which may have led to parental misconceptions.

A definitive cRCT would need to ensure parental communication is clear about the design of the study and the experimental nature of SDD in an infection control regime. Information and training to engage those staff who were unsure or opposed to SDD will be vital to ensure equipoise in the intervention. The time-critical nature of the intervention will need to be considered in the funding model for a definitive cRCT to ensure site staff felt able to deliver workload.

With regards to the ecological outcomes, the consent rate was low for the collection of additional samples. Staff reported challenges to these samples which were perceived as a burden on the patient and staff capacity, particularly during nights shifts, although when consent was obtained, sample collection was very high. An assessment of the ecology impact of SDD is crucial and a future definitive cRCT should consider including admission and discharge ecology monitoring as part of the SDD administration protocol, in order to minimise the burden of consent and ensure antimicrobial resistance is monitored as part of the trial intervention. Traditionally SDD trials have used rectal, rather than perineal swabs to monitor antimicrobial resistance but routine clinical screening uses the latter so we opted for this as a more practical and pragmatic intervention.

## Conclusions

The design of the pilot cRCT was found to be acceptable to both staff and parents in the mixed methods study, suggesting a future definitive PICnIC RCT should use a largely similar design of cluster randomisation with the same eligibility criteria, control and intervention arms, but with adaptations to the SDD paste formulation and dosing regimen, ecology monitoring and consent processes.

### Supplementary Information

Supplementary Tables.

## Data Availability

Requests for de-identified patient data should be submitted to picnic@icnarc.org for consideration by the trial steering committee.
