# Supplementary material for: Selective digestive tract decontamination to prevent healthcare associated infections in critically ill children: the PICNIC multicentre randomised pilot clinical trial
Source: Sci Rep. 2023 Dec 7;13:21668. doi: 10.1038/s41598-023-46232-7 (PMC10709430; doi:10.1038/s41598-023-46232-7)
Supplement: Supplementary file 1 — Supplementary Tables. [file 41598_2023_46232_MOESM1_ESM.docx]

**Supplementary Table 1: Approach to qualitative data analysis**

Supplementary Table 2: Representativeness of participating Paediatric Intensive Care Units

|  |  | **Participating sites** | **UK PICUs** |
| --- | --- | --- | --- |
| Type of PICU |  |  |  |
|  | General | 3 (50%) | 18 (62%) |
|  | General and cardiac | 3 (50%) | 7 (24%) |
|  | Cardiac | 0 (0%) | 4 (14%) |
| PICU beds (intensive care and high dependency) |  |  |  |
|  | <8 | 0 (0%) | 5 (17%) |
|  | 8-11 | 1 (17%) | 1 (3%) |
|  | 12-15 | 2 (33%) | 8 (28%) |
|  | ≥16 | 3 (50%) | 15 (52%) |
| Annual PICU admissions |  |  |  |
|  | <550 | 2 (33%) | 18 (62%) |
|  | 550-749 | 3 (50%) | 6 (21%) |
|  | 750-999 | 0 (0%) | 3 (10%) |
|  | ≥1000 | 1 (17%) | 2 (7%) |

Source PICANet 2022 (PICANet State of the Nation Report https://www.picanet.org.uk/annual-reporting-and-publications/ )

Supplementary Table 3: Representativeness of study patients – baseline characteristics and outcomes

| **Variables** |  | **Potentially eligible patients** | |
| --- | --- | --- | --- |
|  | **Overall** |  |  |
|  |  | **Study PICUs (n=6)** | **All UK PICUs**  **(n=29)** |
|  | **N = 345** | **N = 464** | **N =1730** |
| **Age at admission (months)** |  |  |  |
| Median (IQR) | 6 (32) | 7.0 (42.5) | 5.0 (32.0) |
| Mean (SD) | 30.1 (48.7) | 31.4 (48.7) | 30.2 (49.5) |
| **Age category at admission** |  |  |  |
| <1 year | 197/335 (58.8%) | 269/464 (58%) | 1062/1730 (61%) |
| 1 years | 42/335 (12.5%) | 47/464 (10%) | 168/1730 (10%) |
| 2-4 years | 27/335 (8.1%) | 54/464 (12%) | 184/1730 (11%) |
| 5-9 years | 35/335 (10.4%) | 50/464 (11%) | 138/1730 (8%) |
| 10-16 years | 34/335 (10.1%) | 44/464 (9%) | 178/1730 (10%) |
| **Sex** |  |  |  |
| Male | 209/335 (62.4%) | 280/464 (60%) | 976/1730 (56%) |
| Female | 126/335 (37.6%) | 184/464 (40%) | 754/1730 (44%) |
| **Ethnic category** |  |  |  |
| Asian | 29/335 (8.7%) | 40/464 (9%) | 202/1730 (12%) |
| Black | 15/335 (4.5%) | 27/464 (6%) | 98/1730 (6%) |
| Chinese | 1/335 (0.3%) | 1/464 (0%) | 11/1730 (1%) |
| Mixed | 16/335 (4.8%) | 24/464 (5%) | 68/1730 (4%) |
| White | 195/335 (58.2%) | 262/464 (56%) | 1,028/1730 (59%) |
| Other | 13/335 (3.9%) | 23/464 (5%) | 67/1730 (4%) |
| Unknown | 66/335 (19.7%) | 87/464 (19%) | 256/1730 (15%) |
| **PIM3 predicted risk of PICU mortality (%)** |  |  |  |
| Median (IQR) | 3 (5) | 3.2 (5.3) | 2.2 (4.9) |
| Mean (SD) | 5.4 (9.7) | 6.0 (10.5) | 5.2 (9.0) |
| **Primary diagnosis group** |  |  |  |
| Neurological | 19/335 (5.7%) | 41/464 (9%) | 138/1730 (8%) |
| Cardiac | 78/335 (23.3%) | 109/464 (23%) | 440/1730 (25%) |
| Respiratory | 145/335 (43.3%) | 178/464 (38%) | 738/1730 (43%) |
| Oncology | 4/335 (1.2%) | 8/464 (2%) | 29/1730 (2%) |
| Infection | 43/335 (12.8%) | 48/464 (10%) | 120/1730 (7%) |
| Musculoskeletal | 1/335 (0.3%) | 3/464 (1%) | 20/1730 (1%) |
| Gastrointestinal | 5/335 (1.5%) | 21/464 (5%) | 68/1730 (4%) |
| Other | 20/335 (6.0%) | 29/464 (6%) | 72/1730 (4%) |
| Blood and lymph | 5/335 (1.5%) | 6/464 (1%) | 12/1730 (1%) |
| Trauma | 4/335 (1.2%) | 4/464 (1%) | 21/1730 (1%) |
| Endocrine/metabolic | 6/335 (1.8%) | 9/464 (2%) | 43/1730 (2%) |
| Multisystem | 0/335 (0.0%) | 0/464 (0%) | 3/1730 (0%) |
| Body wall and cavities | 3/335 (0.9%) | 3/464 (1%) | 21/1730 (1%) |
| Unknown | 2/335 (0.6%) | 5/464 (1%) | 5/1730 (0%)) |
| **Died in PICU during admission event** |  |  |  |
| No | 311/335 (92.8%) | 422/464 (91%) | 1,605/1730 (93%) |
| Yes | 24/335 (7.2%) | 42/464 (9%) | 125/1730 (7%) |
| **Length of stay (days)** |  |  |  |
| Median (IQR) | 7 (8) | 8.4 (9.8) | 8.0 (9.7) |
| Mean (SD) | 12.0 (18.4) | 14.4 (20.3) | 14.7 (20.9) |
| **Days free from invasive ventilation** |  |  |  |
| median (IQR)* | 1 (2) | 1 (3) | 1. (4) |

*values censored at PICU discharge, updated figures to day 28 awaited and will be updated once received.

Supplementary Table 4: Effect estimates with 95% confidence intervals for potential outcome measures.

| **Outcome measure** | **Intervention sites** | | **Control sites** | | **Effect estimate (95% CI)** |
| --- | --- | --- | --- | --- | --- |
|  | **Period One** | **Period Two** | **Period One** | **Period Two** |  |
| Healthcare associated infection, n/N (%) | 6/104 (5.8) | 4/56 (7.1) | 16/102 (15.7) | 12/82 (14.6) | 1.31^†^ (0.27, 6.38) |
| Any positive microbiology result, n/N (%) | 62/104 (59.6) | 30/56 (53.6) | 61/102 (59.8) | 49/82 (59.8) | 0.92^†^ (0.34, 2.46) |
| Duration of invasive ventilation (days), [N] | [104] | [56] | [102] | [82] |  |
| Mean (SD) | 8.7 (10.8) | 8.5 (13.2) | 7.7 (6.3) | 6.0 (4.3) | 1.51 (-2.37, 5.38) |
| Median (IQR) | 5 (3, 8) | 5 (4, 10) | 5 (3, 9) | 4 (3, 7) |  |
| Days alive and free of ventilation to day 28†† | [101] | [55] | [100] | [79] |  |
| Mean (SD) | 19.0 (8.8) | 19.9 (7.3) | 18.8 (8.3) | 21.0 (6.6) | -1.57 (-5.05, 1.91) |
| Median (IQR) | 23 (17, 25) | 23 (18, 25) | 23 (15, 25) | 24 (20, 25) |  |
| Length of PICU stay (days), [N] | [104] | [56] | [102] | [82] |  |
| Mean (SD) | 11.5 (13.6) | 11.5 (16.8) | 10.1 (9.3) | 8.3 (7.2) | 1.95 (-3.18, 7.08) |
| Median (IQR) | 7 (4, 13) | 6 (4, 13) | 6 (5, 13) | 6 (4, 10) |  |
| Length of hospital stay (days), [N] | [104] | [54] | [98] | [82] |  |
| Mean (SD) | 26.4 (29.0) | 25.9 (25.2) | 21.7 (28.4) | 21.4 (21.5) | -0.11 (-11.76, 11.55) |
| Median (IQR) | 16 (8, 31) | 15 (8, 39) | 12 (8, 25) | 13 (8, 26) |  |
| PICU mortality, n/N (%) | 7/104 (6.7) | 3/56 (5.4) | 10/102 (9.8) | 4/82 (4.9) | 1.75^†^ (0.27, 11.17) |
| Hospital mortality, n/N (%) | 8/104 (7.7) | 3/54 (5.6) | 13/100 (13.0) | 6/82 (7.3) | 1.41^†^ (0.25, 7.85) |
| 30-day mortality, n/N (%) | 6/103 (5.8) | 3/56 (5.4) | 11/101 (10.9) | 5/82 (6.1) | 1.76^†^ (0.29, 10.72) |

†Odds ratio ^††^ From eligible
